# Segmented nitinol guidewires with stiffness-matched connectors for cardiovascular magnetic resonance catheterization: preserved mechanical performance and freedom from heating

**DOI:** 10.1186/s12968-015-0210-5

**Published:** 2015-11-30

**Authors:** Burcu Basar, Toby Rogers, Kanishka Ratnayaka, Adrienne E. Campbell-Washburn, Jonathan R. Mazal, William H. Schenke, Merdim Sonmez, Anthony Z. Faranesh, Robert J. Lederman, Ozgur Kocaturk

**Affiliations:** National Heart Lung and Blood Institute, National Institutes of Health, Building 10, Room 2c713, Bethesda, MD 20892-1538 USA; Institute of Biomedical Engineering, Bogazici University, Istanbul, Turkey; Department of Cardiology, Children’s National Medical Center, Washington DC, USA

**Keywords:** Interventional cardiovascular magnetic resonance, Heart catheterization, Image-guided intervention, MR safety, MR heating, Medical devices

## Abstract

**Background:**

Conventional guidewires are not suitable for use during cardiovascular magnetic resonance (CMR) catheterization. They employ metallic shafts for mechanical performance, but which are conductors subject to radiofrequency (RF) induced heating. To date, non-metallic CMR guidewire designs have provided inadequate mechanical support, trackability, and torquability. We propose a metallic guidewire for CMR that is by design intrinsically safe and that retains mechanical performance of commercial guidewires.

**Methods:**

The NHLBI passive guidewire is a 0.035” CMR-safe, segmented-core nitinol device constructed using short nitinol rod segments. The electrical length of each segment is less than one-quarter wavelength at 1.5 Tesla, which eliminates standing wave formation, and which therefore eliminates RF heating along the shaft. Each of the electrically insulated segments is connected with nitinol tubes for stiffness matching to assure uniform flexion. Iron oxide markers on the distal shaft impart conspicuity.

Mechanical integrity was tested according to International Organization for Standardization (ISO) standards. CMR RF heating safety was tested in vitro in a phantom according to American Society for Testing and Materials (ASTM) F-2182 standard, and in vivo in seven swine. Results were compared with a high-performance commercial nitinol guidewire.

**Results:**

The NHLBI passive guidewire exhibited similar mechanical behavior to the commercial comparator. RF heating was reduced from 13 °C in the commercial guidewire to 1.2 °C in the NHLBI passive guidewire in vitro, using a flip angle of 75°. The maximum temperature increase was 1.1 ± 0.3 °C in vivo, using a flip angle of 45°. The guidewire was conspicuous during left heart catheterization in swine.

**Conclusions:**

We describe a simple and intrinsically safe design of a metallic guidewire for CMR cardiovascular catheterization. The guidewire exhibits negligible heating at high flip angles in conformance with regulatory guidelines, yet mechanically resembles a high-performance commercial guidewire. Iron oxide markers along the length of the guidewire impart passive visibility during real-time CMR. Clinical translation is imminent.

## Background

Catheterization using cardiovascular magnetic resonance (CMR) is an attractive alternative to X-ray guided procedures because it offers excellent soft-tissue visualization without ionizing radiation, unconstrained imaging planes, and real-time imaging frame rates up to 10 frames per second [1, 2]. Despite these potential advantages, CMR catheterization has not been widely adopted, largely due to the lack of safe, conspicuous, and mechanically satisfactory guidewires [3–5]. Commercial guidewires contain long metallic components for mechanical performance, X-ray conspicuity, and affordability. Unfortunately commercial guidewires risk heating during CMR because of standing wave formation along the conductive parts longer than a quarter wavelength at the resonant frequency, which corresponds to approximately 12 cm in humans at 1.5 T [6].

Numerous investigators have proposed CMR-safe guidewire designs [4, 5, 7, 8], some of which have even been tested in patients [9, 10]. Active designs are conspicuous because they incorporate antenna receiver elements, and they attempt to reduce heating by incorporating RF chokes or transformers along the shaft. Active designs proposed to date are mechanically tethered at their proximal end, limiting their versatility [11], or have other mechanical limitations [12, 13]. Passive-designs rely on intrinsic material properties for device visualization, and usually incorporate non-metallic (polymer) components to eliminate RF induced heating. However they consequently have inferior mechanical properties compared to their metallic counterparts, such as low torque-response, inadequate column strength to deliver catheter devices, reduced kink resistance, and difficulty navigating tortuosity [14].

We describe a simple, practical, inexpensive guidewire design that is intrinsically safe for use in CMR. We connect short insulated metallic segments, which are incapable of RF-induced heating at 1.5T, into a guidewire. However, interconnected short rods would flex non-uniformly, like a “folding cane”. Therefore, we connect insulated metallic segments using stiffness-matched connectors to provide uniform flexion similar to conventional long metallic guidewires. The guidewire incorporates passive iron oxide markers to provide CMR conspicuity. In this paper we describe initial bench top tests for guidewire torquability, trackability, and column strength. We demonstrate freedom from RF-induced heating in vitro and in vivo. Finally, we demonstrate clinical applicability of the guidewire by performing CMR guided cardiac catheterization in vivo in swine.

## Methods

### MR-safe segmented Nitinol-Core guidewire design

**Fig. 1:**
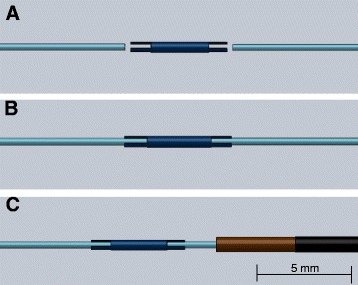
NHLBI passive guidewire design schematic. **a** The core of the guidewire consists of 10 cm nitinol rod segments (light blue) joined by 5 mm nitinol connectors (dark blue) for stiffness matching. **b** Each nitinol connector has notched ends that join two inserted nitinol rods. A full-length (260 cm) guidewire has 25 such connectors. **c** The segmented-core is jacketed with a braided polymer jacket (brown) for support. A soft polymer (black) is melted over the jacketed core as a final layer

**Fig. 2:**
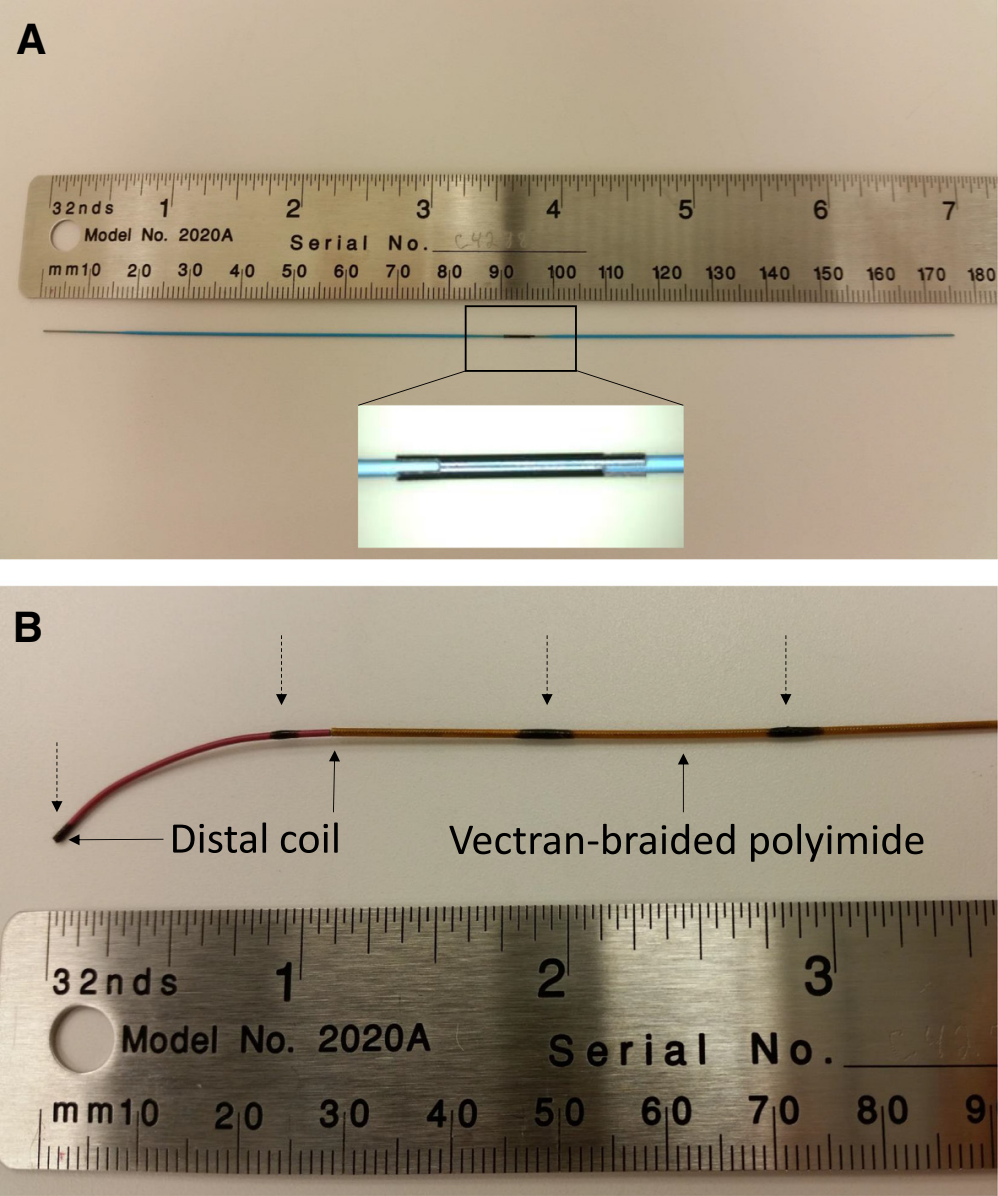
Components of the NHLBI passive guidewire. **a** This photograph shows two Pebax-coated nitinol segments are joined by a nitinol connector. **b** The distal tip incorporates a non-resonating coil for mechanical flexion, which is wrapped over the shaped and tapered nitinol rod segment. The distal and proximal ends of the coil are indicated by arrows. Iron oxide markers (dashed arrows) are painted over the tip coil and the Vectran-braided polyimide tubing prior to the application of the final polymer jacketing layer

A nitinol guidewire with typical exchange-length geometry (0.035” outer diameter and 260 cm length) was constructed using the segmented design (Fig. 1). Each segment is 10 cm long to prevent standing wave formation and avert RF-induced heating at 1.5 T [6]. Each segment consists of a 0.014” nitinol rod coated with a thin film of parylene for electrical insulation, and jacketed with thermoplastic polymer (Pebax, Zeus Inc, Orangeburg, SC) to impart a consistent profile between the rods and the connectors. The insulated rod segments are inserted and secured into the connector tubes using medical grade UV-cured adhesive (Dymax Corporation, Torrington, CT) to create the non-conducting metallic core (Fig. 2). The connectors are 5 mm long, laser-cut nitinol tubes. Insulation faults, which would risk RF-induced heating, are prevented by insulating both ends of each connector tube. The rod-connector subassembly is surrounded by polymer (Vectran) fiber-braided polyimide tubing (0.026” inner diameter, 0.032” outer diameter) to augment pushability, torque response, kink resistance, and dielectric properties.

The distal nitinol rod segment tapers from 0.014” to 0.005” over 5 cm. A 3 cm long coil (MP35N alloy, Heraeus, MN) is mounted onto the distal tip to enhance flexion and recoil (Fig. 2). This tip is jacketed with non-braided thermoplastic polymer for softness and flexibility. Pre-shaped distal tip configurations are available including J- and angled.

Iron oxide powder (Sigma Aldrich, St. Louis, MO) is blended with a UV-cure adhesive (Dymax Corporation) and applied onto the guidewire for passive visualization, at the distal and proximal end of the distal tip coil, and every 10 cm thereafter. The markers underlie a final layer of thermoplastic jacketing to avoid blood contact and to bring the outer diameter of the guidewire to 0.035”.

### Mechanical tests

**Fig. 3:**
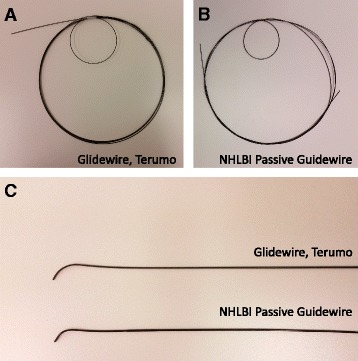
The NHLBI passive guidewire prototype compared with a Terumo *Glidewire*. **a** A 260-cm commercial Terumo Glidewire, and (**b**) a 260-cm NHLBI passive segmented nitinol-core guidewire are tightly coiled. Both exhibit similar bending curvatures. **c** The tip configurations are similar

We tested the NHLBI passive guidewire alongside a high performance nitinol based commercial (non-segmented) comparator (*Glidewire Standard* GR3509, Terumo, Tokyo). Both are depicted in Fig. 3. Rod-connector subassemblies and the coiled distal tip subassemblies (*n* = 5 each) were tested for tensile breakage force against a stationary jaw. The industry standard minimum tensile strength for an 0.035” guidewire is 5N [15].

Flexibility of the tapered, coiled distal tip was measured as the force required to deflect 45° and 60° at 5 mm, 10 mm, and 20 mm from the tip using an Intravascular Device Testing Equipment (IDTE 2000, Machine Solutions Inc., Arizona) according to US Food and Drug Administration (FDA) guidance [16] .

Torque response and pushability tests were performed in a custom vascular model of a left heart cardiac catheterization trajectory from femoral artery across the aortic arch and aortic valve into the left ventricle. The number of rotations applied at the proximal end was plotted against the number of rotations transmitted to the distal end to evaluate torque transmission inside the vascular model. Force required to advance 65 cm through this trajectory at a constant speed was measured to assess guidewire pushability [16].

### *In Vitro* RF-induced heating tests

Heating tests were performed in a 1.5T MR system (Aera, Siemens, Erlangen, Germany) using a balanced steady-state free precession (bSSFP) pulse sequence under typical real-time CMR operating conditions (TR/TE, 2.9/1.4 ms; flip angle, 45°, bandwidth, 1000 Hz/pixel; matrix, 192 × 108; FOV, 300 × 300 mm; GRAPPA Factor 2). The tests were performed under high-flip-angle (75°) conditions to induce a high Specific Absorption Rate (SAR).

In vitro RF-induced heating tests used an ASTM 2182 phantom [17]. Temperature was measured using a fiberoptic temperature probe (OTG-M170, Opsens Inc., Canada) with a thermal resolution of 0.1 °C and an accuracy of 0.3 °C. The probe was fed through a polyimide channel (0.009” ID, 0.011” OD) affixed alongside the guidewire using heat shrink tubing (Advanced Polymers, Salem, NH). The guidewire tip was positioned at the designated hot-spot of the phantom (6 cm depth; 11 cm off-center). The temperature probe channel extended 1cm beyond the guidewire tip.

Temperature was recorded continuously for 30 s before initiating CMR scanning, for 30 s after initiating CMR scanning, for 60 s after probe withdrawal to the guidewire tip, and then while the probe was withdrawn.

### *In Vivo* RF-induced heating tests

Animal experiments were approved by the NHLBI Animal Use and Care Committee and performed according to contemporary NIH standards, in swine under general anesthesia after percutaneous femoral artery and vein access. Four animals underwent RF-induced heating tests (weight = 24–63 kg).

In vivo heating data were acquired through a fiber optic probe positioned at the distal tip of the guidewire to monitor RF-induced temperature rise in each animal. Probe, rectal core body temperature, and their instantaneous difference were recorded continuously. Upon femoral access through an introducer sheath (Pinnacle Terumo, 5 F), the guidewire was advanced to the aortic arch while acquiring 60-s stationary temperature data at various insertion lengths inside the body. Upon reaching the arch, a temperature-probe pull-back was performed by retracting the probe while holding the guidewire stationary to evaluate heating along the length of the guidewire.

### Guidewire conspicuity under CMR

In vitro images were obtained in a phantom prepared according to ASTM F2119-07 “Standard Test Method for Evaluation of MR Artifacts from Passive Implants” [18] to assess guidewire conspicuity under CMR. The two markers tested used iron oxide powder consisting of 97 % or 99.99 % purity (trace metal-basis, product numbers: 637106, 518158, Sigma Aldrich). Conspicuity of the two markers was evaluated on GRE images (TR/TE, 612/10 ms; thickness, 5 mm; FOV, 300 × 300 mm; matrix, 128 × 128). Contrast-to-noise ratio (CNR) between the markers and phantom was calculated according to the difference method [19], and the size of The marker susceptibility artifacts was evaluated according to ASTM standard F2119-07 [18].

In vivo images were acquired using an interactive, real-time bSSFP sequence (TR/TE, 2.88/1.44 ms; thickness, 6 mm; FOV, 350 × 350 mm; matrix, 192 × 144) typically used to for CMR catheterization at our institution. This sequence was chosen over GRE by the operators because it is faster and provides higher overall Signal-to-Noise Ratio, even though the size of the susceptibility artifacts may be larger with unbalanced gradient echo techniques [20].

### *In Vivo* heart catheterization

Left heart catheterization was performed on seven swine, including the four used for heating experiments. The NHLBI guidewire was introduced through the femoral artery and navigated without a support catheter from the femoral artery around the aortic arch and across the aortic valve into the left ventricle to assess guidewire pushability, steerability, and torquability. The guidewires were re-sterilized and re-used after catheterization cases to evaluate durability.

## Results

### Mechanical tests

**Fig. 4:**
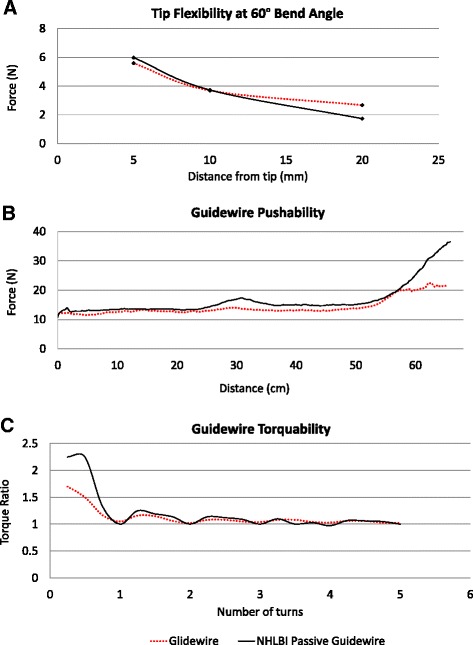
Mechanical tests. The NHLBI passive guidewire (black) and the Glidewire (red) showed comparable mechanical characteristics. **a** Force required to deflect the tip to a selected bend angle. **b** Pushability test depicts the force to advance the guidewire through a vascular phantom. **c** Torquability inside the vascular phantom indicates torque ratio (number of rotations observed at the distal end of the guidewire per rotation applied at the proximal end) of the two guidewires

The force required to separate two adjoined segments is 11.34 ± 2.14 N, which surpasses the 5 N requirement of the ISO standard [15]. When the samples were jacketed with a Vectran-braided outer layer, the tensile force required to break the subassembly increased three-fold (30.97 ± 0.20N). By comparison, the *Glidewire* comparator outer layer breaks at forces above 30 N. The force required to detach the tip coil from the distal core segment of the NHLBI passive guidewire was 19.24 ± 1.30 N (*n* = 5). We found that the distal tip exhibited similar flexibility characteristics and the overall torque response compared well to the *Glidewire* (Fig. 4).

### *In Vitro* RF-induced heating tests

**Fig. 5:**
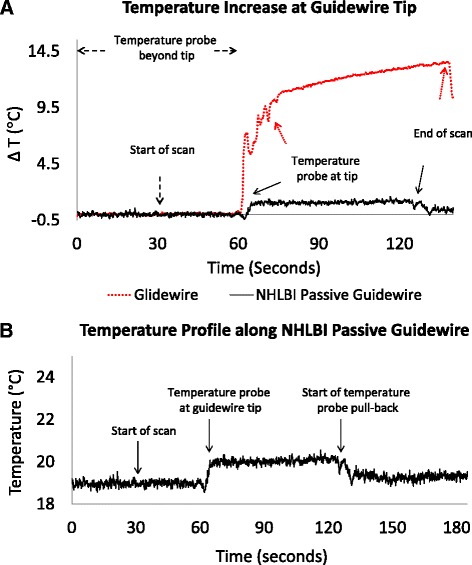
In vitro temperature measurements. **a** Temperature at the tip of the NHLBI passive guidewire (black) and the commercial Glidewire comparator (red) during 60 s bSSFP CMR scans at a flip angle of 75°. **b** Pullback of the temperature probe alongside the NHLBI passive guidewire. After baseline steady state, scanning begins (at t = 30 s), then the probe is withdrawn from beyond the guidewire tip back to the guidewire tip (at t = 60 s). The probe remains at the tip, during which time the temperature rises by 1.2 °C. Finally the probe is pulled back further (beginning at t = 120 s) indicating that the temperature rise is confined to the tip

In vitro the temperature change measured at the tip of a 260 cm long *Glidewire* is 13 °C compared with 1.2 °C observed at the tip of the NHLBI passive guidewire at the end of a 60-s scan (Fig. 5) at a 75° flip angle (scanner-reported whole body SAR; 0.6 W/kg). Probe pull-back tests show that the maximum temperature increase occurs at the tip of the guidewire, and that there is no heating at the connectors along the shaft (Fig. 5).

### Guidewire visibility under CMR

**Fig. 6:**
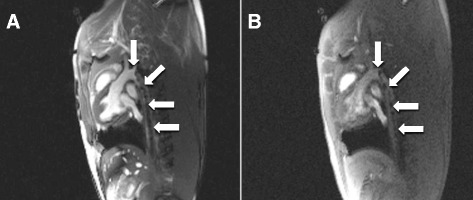
CMR of the NHLBI passive guidewire in vivo. The effect of various imaging sequences on in vivo guidewire conspicuity is illustrated. **a** During bSSFP CMR the iron-oxide susceptibility markers are conspicuous as the guidewire is advanced retrograde through the descending thoracic aorta. The signal-to-noise ratio is higher for images acquired using bSSFP [TR/TE, 2.88 ms/1.44 ms] compared with (**b**) GRE sequences with similar TE [TR/TE, 4.87 ms/2.18 ms]

The iron oxide articles with higher purity (99.99 %) provided a slightly higher CNR (172 vs 159) and acceptable susceptibility artifact (2 cm vs 1.2 cm) and were chosen as markers. These iron markers rendered the guidewires conspicuous in vivo during left heart catheterization in swine, using both SSFP and GRE pulse sequences (Fig. 6).

### *In Vivo* catheterization and heating experiments

Left heart catheterization was successful in all 7 animals. The operators found the NHLBI passive guidewire had similar mechanical functionality to commercial guidewires for heart catheterization, even after re-sterilization and re-use. This included slight loss of tip shape.

**Fig. 7:**
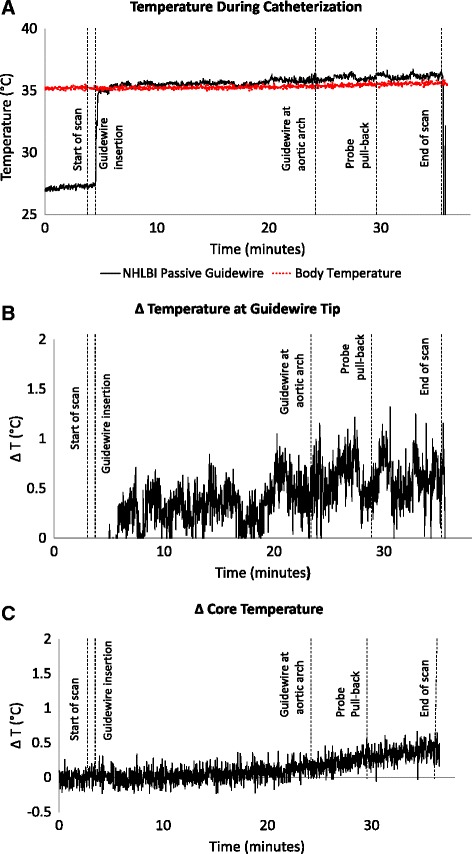
Temperature during left heart catheterization in swine. The panels depict three simultaneous tracings during bSSFP with a flip angle of 45°. First scanning begins, then the guidewire is advanced retrograde to the aortic arch, and then a temperature probe is withdrawn alongside the guidewire in order to measure tip and shaft temperature. **a** shows the guidewire temperature probe (black) and simultaneous core body temperature (red). On a narrower scale, (**b**) shows the instantaneous difference between the guidewire and core body temperature, while (**c**) shows the core body temperature rise during CMR. The guidewire temperature rise was negligible

In vivo RF-induced heating was evaluated during left heart catheterization in four swine (weight = 43 ± 17.6 kg) at a scanner-reported peak whole-body SAR (whole body SAR) of 1.6 W/kg over 30 min. Temperature at the distal tip of the guidewire showed no detectable heating during advancement (Fig. 7). Similarly, a temperature probe withdrawn alongside the shaft of the guidewire in vivo showed no detectable heating (Fig. 7). The averaged maximum temperature increase was 1.1 ± 0.3 °C above body temperature at a thermal resolution of 0.1 °C and accuracy of 0.3 °C. The greatest temperature difference between the body temperature and the temperature measured at the tip was 1.3 °C during all four experiments, while the maximum body temperature increase was 0.6 ± 0.1 °C.

The body temperature of a separate animal was monitored while scanning without a guidewire. In the absence of a guidewire, the body temperature increased from 35.9 °C to 36.4 °C (ΔT_body_ = 0.5 °C) at a 45^o^ flip angle, and from 36.3 °C to 37.3 °C (ΔT_body_ = 1 °C) at a 75° flip angle (scan time = 35 min).

## Discussion

We describe a novel segmented-core nitinol guidewire for use in CMR that is intrinsically safe by design and that incorporates iron oxide nanoparticle markers for passive visualization. This NHLBI passive guidewire has two key features. One is that all metallic components are less than one-quarter wavelength of RF transmission in the body at 1.5 T (<10 cm). As a result, standing waves are unable to form, and in turn RF-induced heating is averted. Second, the short metallic segments are interconnected using insulated couplers to create a continuously flexible nitinol system that otherwise behaves mechanically like a conventional guidewire. Specifically, the coupled segments resist separation when exposed to high tensile forces, comparable to non-segmented commercial guidewires.

Several CMR guidewire designs have been proposed that aim to mitigate RF-induced heating [4, 5, 7–9, 21]. Unfortunately, passive guidewire designs lack the torquability, trackability, and support properties required for interventional catheterization due to their polymer-only construction [8, 14, 22, 23] while active metallic device designs currently do not allow catheter exchanges because of fixed instrumentation at the hub [11]. In contrast, the NHLBI passive guidewire performs similarly to a high performance commercially available guidewire in vitro and in vivo.

Guidewire heating was minimal both in vitro and in vivo. The maximum instantaneous temperature increase at the guidewire tip was 1.3 °C in vivo at a peak whole body SAR of 1.6 W/kg over 30 min, although the time-averaged rise was below 1 °C (Fig. 7). Of note, during this protracted CMR, the animal core body temperature increased by 0.5 °C independent of the guidewire.

The controlled susceptibility artifacts created by the iron oxide markers placed along the guidewire shaft were sufficient for tip tracking and left heart catheterization. We found the higher purity iron formulation to generate larger artifacts with slightly higher CNR using gradient echo CMR, which was preferable for this application.

A notable feature of the NHLBI passive guidewire is that because it is intrinsically safe by design, it may permit high contrast images without risking heating at higher flip angles. This specific guidewire was designed to operate at 1.5 T. Shorter non-resonant segments would be required at higher field strengths.

### Limitations

A segmented-core design risks fracture or separation of segments. The NHLBI passive guidewire incorporates a reinforcing polymer braid over the core and connectors, which mitigates this failure mode. In our experience, guidewire integrity was maintained despite extensive re-use, but tip shape was compromised, similar to commercial guidewires.

Heating tests were performed without a catheter, which might alter insulation, RF-induced heating properties, and blood-flow-induced cooling. RF-induced heating is known higher within transfemoral vascular introducer sheaths, which are often located in high-E-field regions of the scanner [11]. However, we did not observe higher temperatures using the NHLBI passive guidewire inside vascular introducer sheaths. Temperature measurements using physical probes suffer geometric constraints and may alter insulation properties. Alternative approaches would be attractive, however we have found fast T1 thermometry [24, 25] unsuccessful because of signal dephasing near the tip of our guidewire, both in vitro and in vivo, although it would be an attractive method to assess heating in the surrounding tissue in vivo.

The NHLBI passive guidewire suffers from typical limitations of susceptibility-artifact-based visualization. Iron-induced susceptibility artifacts are non-specific and cannot exploit the positive-contrast afforded by, for example, gadolinium-filled balloons [1]. Multi-echo imaging [26] and off-resonance reconstruction [27] may in part compensate for these shortcomings.

## Conclusion

We describe a novel 0.035” CMR guidewire that by design fulfills mechanical and MR-safety requirements imposed by international standards. RF-induced heating is successfully constrained below the recommended 2 °C limit during typical CMR catheterization scanner conditions, by segmenting the conductive nitinol core of the guidewire.

Joining the segments using stiffness-matched connectors achieves comparable mechanical characteristics to a high-performance commercially available nitinol guidewire while retaining the electrical isolation of the individual short segments. The controlled image artifacts created by iron oxide markers incorporated onto the guidewire shaft for passive visualization enables cardiac catheterization in a large animal model.
